# EGFR-TKIs耐药继发*MET*基因扩增肺鳞癌1例报告并文献复习

**DOI:** 10.3779/j.issn.1009-3419.2024.106.32

**Published:** 2024-11-20

**Authors:** Yalan LIU, Peng CHEN, Xinfu LIU

**Affiliations:** ^1^422000 邵阳，湖南省邵阳市中心医院肿瘤内科（刘亚岚，刘新福）; ^1^Department of Oncology, The Central Hospital of Shaoyang, Shaoyang 422000, China; ^2^300060 天津，天津医科大学肿瘤医院肺部肿瘤内科，国家肿瘤临床医学研究中心（刘亚岚，陈鹏）; ^2^Department of Thoracic Oncology,Tianjin Medical University Cancer Institute and Hospital, National Clinical Research Center for Cancer, Tianjin 300060, China

**Keywords:** 肺肿瘤, EGFR-TKIs, MET, 肺鳞癌, Lung neoplasms, EGFR-TKIs, MET, Squamous cell lung cancer

## Abstract

肺腺癌患者目前已常规开展表皮生长因子受体（epidermal growth factor receptor, EGFR）的基因检测，而部分小样本的不吸烟的女性肺鳞癌患者也有EGFR突变的可能，肺癌靶向治疗的快速发展为这一类患者增加了靶向治疗的机会。然而肺鳞癌患者行靶向治疗期间出现耐药是影响后续治疗的重要因素。靶向治疗获得性耐药有多种机制，间充质细胞上皮转化因子（mesenchymal-epithelial transition factor, MET）信号通路的改变是其中常见的耐药机制之一。目前，一些MET的选择性酪氨酸激酶抑制剂（tyrosine kinase inhibitors, TKIs）已获批用于伴 MET基因14外显子跳跃突变的非小细胞肺癌，如谷美替尼、塞沃替尼、特泊替尼、卡马替尼等，而针对继发MET基因扩增的药物尚处于临床试验阶段。本文通过回顾性分析1例EGFR-TKIs耐药继发MET基因扩增女性肺鳞癌患者的临床资料，并复习相关文献，探讨如何优选具有EGFR突变的肺鳞癌患者的治疗方案，为该类患者的诊疗提供临床借鉴及参考。

## Abstract (en)

With the rapid development of epidermal growth factor receptor (EGFR) gene testing of lung adenocarcinoma patients has been routinely carried out, EGFR mutations are also possible for some small samples of non-smoking female lung squamous cell carcinoma patients. This increases the opportunity for targeted therapy for this group of patients. However, drug resistance in patients with lung squamous cell carcinoma during targeted therapy is an important factor affecting subsequent treatment. There are multiple mechanisms of acquired drug resistance in targeted therapy, and the alteration of mesenchymal-epithelial transition factor (MET) signaling pathway is one of the common mechanisms of drug resistance. At present, some selective tyrosine kinase inhibitors (TKIs) of MET has been approved for non-small cell lung cancer with MET gene 14 exon skipping mutation, such as Glumetinib, Savolitinib, Tepotinib, Capmatinib, etc. Drugs that target secondary MET amplification are still in clinical trials. This paper retrospectively analyzed the clinical data of a female patient with EGFR-TKIs resistant secondary MET amplified squamous cell lung cancer, and reviewed relevant literature to explore how to optimize the treatment of lung squamous cell carcinoma patients with EGFR mutation, so as to provide clinical reference for the diagnosis and treatment of such patients.

2024年4月国际癌症研究机构（International Agency for Research on Cancer, IARC）公布了全球各地区在2022年的癌症统计数据^[1]^，肺癌的发病率及死亡率仍居全球癌症前列。随着对肺癌致病机制研究的不断深入，非小细胞肺癌（non-small cell lung cancer, NSCLC）中的驱动基因如表皮生长因子受体（epidermal growth factor receptor, EGFR）、间充质细胞上皮转化因子（mesenchymal-epithelial transition factor, MET）、肉瘤致癌因子（ROS proto-oncogene 1, ROS1）、间变性淋巴瘤激酶（anaplastic lymphoma kinase, ALK）、v-raf鼠肉瘤病毒癌基因同源体（v-Raf murine sarcoma viral oncogene homolog, BRAF）、神经营养因子受体酪氨酸激酶（neurotrophin receptor kinase, NTRK）及大鼠肉瘤癌基因（Kirsten rat sarcoma viral oncogene, KRAS）等相继被发现^[2]^。相关靶向药物的研发及上市应用为NSCLC的治疗带来了曙光，尤其是EGFR-酪氨酸激酶抑制剂（tyrosine kinase inhibitors, TKIs）和ALK-TKIs的使用让一部分晚期肺癌患者达到“去化疗”状态，明显改善了他们的生活质量^[3]^。不同于晚期肺腺癌有诸多驱动基因指导靶向治疗，肺鳞癌患者的治疗手段有限，化疗仍属于一线治疗方案的基石。对于部分老年肺鳞癌且抗拒化疗的患者，若基因检测能发现敏感突变，应用靶向药物治疗也是一种可选策略。目前已有临床研究^[4]^对于EGFR-TKIs耐药后的靶向药物进行探索，也为获得性耐药患者提供了一些治疗机会。本文在获得患者家属知情同意下，报道2024年3月邵阳市中心医院收治的1例EGFR-TKIs耐药继发MET基因扩增的女性肺鳞癌病例的临床资料，并复习相关文献资料，为此类患者的临床诊疗及用药筛选提供新思路。

## 1 病例资料

患者，女，72岁，身高156 cm，体重45 kg，因“确诊左肺鳞癌1年9个月，左侧胸背部疼痛半月”于2024年3月20日就诊于湖南省邵阳市中心医院肿瘤内科。既往无基础疾病及恶性肿瘤家族史，无吸烟饮酒史。患者自诉2022年6月因发现左肺占位于长沙某上级医院就诊，6月18日正电子发射型计算机断层显像（positron emission tomography/computed tomography, PET/CT）显示，左肺上叶软组织结节伴瘤周结节，代谢局限性增高，考虑肺癌可能性大，最大肿块测量约70 mm•69 mm；右肺结节，左侧肺门、纵隔多发淋巴结代谢局限性增高，考虑为肺恶性肿瘤转移所致，需结合病理检查。完善支气管镜结果显示：（左上叶上部）非角化型鳞癌（图1），免疫组化结果：甲状腺转录因子-1（thyroid transcription factor-1, TTF-1）（-），新天冬氨酸蛋白酶A （new aspartic proteinase, NapsinA）（-），细胞程序性死亡配体1（programmed cell death ligand 1, PD-L1）（E1L3N）肿瘤细胞阳性比例评分（tumor proportion score, TPS）<1%，细胞角蛋白（cytokeratin, CK）5/6（+），CK7（+），P40（+）。初诊分期为cT3N2M1a IVA期，原发肿瘤体积较大并肺内、纵隔淋巴结转移，经胸外科医师会诊评估无手术指征，该院肿瘤科医生建议以化疗为主的内科综合治疗。患者家属考虑其高龄担心无法耐受副反应，故拒绝行全身化疗，要求完善基因检测寻找靶向药物。患者在该院病理科行肺癌EGFR/ALK/ROS1/MET/RET/BRAF/NTRK基因检测示：EGFR第21号外显子错义突变p.L858R，丰度81.82%，其余各项基因检测均为阴性（表1）。由于存在敏感基因突变，故患者从2022年6月下旬开始口服奥希替尼（80 mg）每日一次进行靶向治疗，每隔2-3个月在当地医院复查提示病情好转，自诉原发肿瘤最小达到35 mm•30 mm，疗效评估为部分缓解（partial remission, PR）。2023年1月下旬患者无诱因出现左侧胸背疼痛不适，数字评分量表（numeric rating scale, NRS）评分为3分，活动后明显，无明显胸闷气促，无恶心呕吐，无腹胀腹痛等。2023年2月1日患者来我院门诊完善胸部+上腹部CT检查显示：（1）左肺上叶占位灶，考虑周围型肺癌伴纵隔淋巴结转移，请结合临床；（2）右肺多发小结节，转移可能；（3）肝左右交界区血管瘤，肝多发囊肿。CT报告测量肿块大小约63 mm•49 mm，根据实体肿瘤疗效评价标准（Response Evaluation Criteria in Solid Tumors, RECIST）1.1版，提示病情进展（progressive disease, PD）。患者入院后于2023年2月17日行经皮肺穿刺活检，病理结果为（左肺穿刺活检）非角化型腺样鳞癌（图2）。免疫组化结果：CK5/6（+），P40（+），CK7（+），TTF-1（-），NapsinA（-），突触素（synaptonin, Syn）（-），嗜铬粒蛋白A（chromogranin A, CgA）（-），EGFR（+），肿瘤增殖标志物Ki-67（marker of proliferation Ki-67, Ki-67）（+70%）。进一步完善肺癌驱动基因检测结果为EGFR第21号外显子错义突变p.L858R（丰度92.02%）合并MET高水平扩增（拷贝数=11）（表1）。家属慎重考虑后仍拒绝全身化疗，2023年3月2日开始口服奥希替尼（80 mg）每日一次联合赛沃替尼（300 mg）每日一次进行靶向治疗，期间于门诊定期复查提示肿瘤持续缩小，疗效评估为PR（图3）。因患者再次出现左侧胸背疼痛于2024年3月20日入我院就诊，行羟考酮缓释片止痛等对症处理后好转出院，期间未停止口服靶向药物。该患者一线服用奥希替尼的无进展生存期（progression-free survival, PFS）为9个月，二线服用奥希替尼联合赛沃替尼的PFS达到12个月。在口服奥希替尼联合赛沃替尼期间，患者每隔2-3个月监测血常规、血脂及肝肾功能未见明显异常，仅出现轻度的食欲减退及口腔黏膜炎，不影响日常生活，至患者末次复查（2024年1月8日）时均未发现靶向药物所致的心肺功能异常。根据最新随访结果，患者因“双肺肺炎”（图4）致反复发热（体温最高39.8 ^o^C）、低氧血症在当地医院救治无效于2024年4月18日病逝，总生存期（overall survival, OS）为21个月。结合病史考虑患者死因为感染因素所致的急性呼吸衰竭可能性较大。

**图1:**
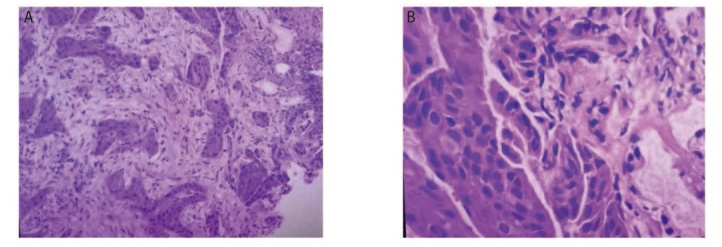
患者第1次病理检查结果（非角化型鳞癌）。苏木精-伊红染色法，A：×100；B：×400。

**表1:** 患者两次基因检测结果比较

Gene name	Mutation name	1^st^ Result	2^nd^ Result	Medication tips
EGFR	Exon 19 deletion	Negative	Negative	
	L858R/G719X/L861Q/S768I	p.L858R (81.82%)	p.L858R (92.02%)	Gefitinib, Erlotinib, Osimertinib
	T790M	Negative	Negative	
	Exon 20 insertion	Negative	Negative	
ALK	Fusion	Negative	Negative	
ROS1	Fusion	Negative	Negative	
BRAF	V600E	Negative	Negative	
KRAS	G12X/G13/Q61X/A146X/K117N	NA	Negative	
ERBB2	Oncogenic mutation	NA	Negative	
RET	Fusion	Negative	Negative	
MET	Copy number variation/exon 14 skipping mutation	Negative	Copy number=11 (high level amplification）	
NTRK1/2/3	Fusion	Negative	Negative	

EGFR: epidermal growth factor receptor; ALK: anaplastic lymphoma kinase; ROS1: ROS proto-oncogene 1; BRAF: V-Raf murine sarcoma viral oncogene homolog B; KRAS: Kirsten rat sarcoma viral oncogene; ERBB2: Erb-B2 receptor tyrosine kinase 2; RET: rearranged during transfection; MET: mesenchymal-epithelial transition factor; NTRK: neurotrophin receptor kinase; NA: not available.

**图2:**
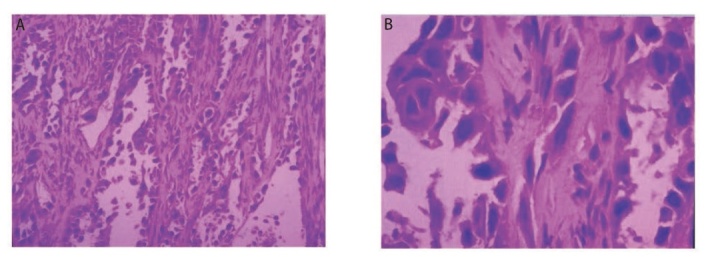
患者第2次病理检查结果（非角化型腺样鳞癌）。苏木精-伊红染色法，A：×100；B：×400。

**图3:**
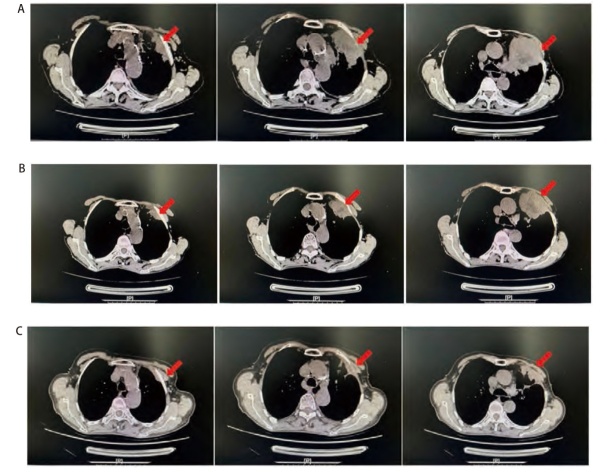
患者服用奥希替尼+赛沃替尼期间影像学检查表现。A: 患者2023年2月27日胸部CT提示病灶大小为63 mm×49 mm；B: 患者2023年4月25日胸部CT提示病灶大小为43 mm×38 mm；C: 患者2024年1月8日胸部CT提示肿瘤形态不规则，较前明显缩小。图中红色箭头所指为原发肺部肿瘤。

**图4:**
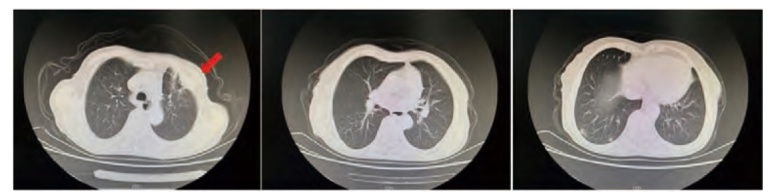
患者2024年4月12日胸部CT示双肺渗出性病灶，左肺占位较前无改变。图中红色箭头所指为原发肺部肿瘤。

## 2 讨论

### 2.1 分子靶向治疗在EGFR突变肺鳞癌中的研究进展

2023年发布的中华医学会肺癌临床诊疗指南指出，由于肿瘤的异质性，经过小标本活检诊断的肺鳞癌可能存在未被检测到的混合腺癌成分。因此对于不吸烟、经小标本活检诊断或混合腺癌成分的肺鳞癌患者，建议进行EGFR基因检测^[5]^。早在2019年，中国临床肿瘤学会（Chinese Society of Clinical Oncology, CSCO）指南就进行了更新：增加并限定不吸烟、经小标本活检诊断鳞癌或混合腺癌成分的患者进行EGFR突变、ALK融合及ROS1融合检测（2A类证据），作为II级推荐^[6]^。该例女性肺鳞癌患者在初诊时病理结果为非角化型鳞癌，在EGFR-TKIs耐药后再次活检结果为非角化型腺样鳞癌，虽然病理性质未出现转化，但由于患者为亚裔女性，无吸烟史，检测采用的是小样本活检标本，不排除大体肿瘤中混杂腺癌成分，这也可能是患者首次基因检测出现敏感驱动基因突变的原因之一。

相比亚裔不吸烟女性肺腺癌有高达50%的EGFR突变率，EGFR在肺鳞癌中的突变率则很低。Sun等^[7]^的研究中报道了1359例肺鳞癌患者中仅有94例患者检测出EGFR突变，阳性率仅为6.9%。除了常见的敏感突变外，这些患者中还检测出一种特殊的突变类型，即EGFR III型突变体，其发生概率为5.0%-8.0%，这类患者使用靶向药物治疗的生存获益远不如敏感突变的患者。4项关于靶向治疗EGFR突变的非腺癌患者疗效的研究^[8⇓⇓-11]^显示，靶向药物对EGFR突变肺鳞癌患者的疗效比肺腺癌要差，有效率为26.7%-38.0%，中位PFS为3.1-3.98个月；一项包含269例EGFR敏感突变腺癌患者的研究^[12]^显示，靶向治疗有效率为77.7%，中位PFS为11.27个月。总体而言，肺鳞癌EGFR突变型的总反应率要高于野生型，分别为25%和9.1%。LUX- Lung 8研究^[13]^结果显示，在既往接受过一线化疗的非选择性的鳞癌患者中，二线治疗使用阿法替尼对比厄洛替尼其OS分别为7.9和6.8个月，风险比为0.81[95%可信区间（confidence interval,CI）：0.69-0.95,P=0.0077]，PFS分别为2.6和1.9个月，风险比为0.81（95%CI: 0.69-0.96, P=0.0103），阿法替尼较厄洛替尼组生存期有明显提高，具有统计学差异。基于该项研究结果，国家药品监督管理局（National Medical Products Administration, NMPA）于2017年2月批准阿法替尼二线治疗晚期非选择性肺鳞癌患者^[14]^。在不同的回顾性研究中EGFR-TKIs对肺鳞癌的疗效有所差异，这可能与研究纳入样本量、基因检测方法、靶向药物的选择和患者的临床基本特征密切相关。肺鳞癌患者在一线EGFR-TKIs治疗耐药后，仍可考虑行二次活检明确耐药原因，如出现继发突变且患者无法耐受化疗副反应，有条件者可选择相应靶点的药物进一步治疗。在二线靶向治疗过程中需要定期监测药物不良反应及评估疗效，一旦出现病情快速进展则及时调整治疗方案，排除禁忌首选含铂双药化疗。由于免疫检查点抑制剂（immune checkpoint inhibitors, ICIs）在肺癌治疗中的迅速发展，在未经选择的晚期肺鳞癌或野生型肺鳞癌患者中更多采用免疫或免疫联合化疗作为首选治疗方案^[15]^，若检测属于EGFR野生型患者，也不能从EGFR-TKIs中获益。

### 2.2 EGFR突变合并MET扩增的研究进展

近二十年随着国内外EGFR-TKIs不断上市，第一代、二代及三代EGFR-TKIs均得到CSCO指南的一致推荐，但不同药物的作用机制略有差异^[16]^。第一代EGFR-TKIs如吉非替尼能可逆地与EGFR结合，阻断细胞内的信号传导。第二代EGFR-TKIs如阿法替尼能作用于多个靶点，与EGFR的结合是不可逆的，使所有肿瘤细胞表达人表皮生长因子受体（human epidermal growth factor receptor, HER）家族同源或异二聚体信号传导受抑制。相比较于前两代药物，第三代EGFR-TKIs如奥希替尼可以选择性抑制EGFR突变，对于第20号外显子T790M突变患者仍有效果，且副作用小于第二代药物，已成为目前指南及专家共识优选的药物^[17]^。虽然EGFR-TKIs为晚期NSCLC患者延长了OS，但仍有20%-30%的EGFR突变的患者靶向治疗效果不尽人意，甚至有部分患者在治疗初期就出现快速进展，机制之一为合并原发或继发驱动基因改变^[18]^。作为原发驱动基因，EGFR-TKIs耐药合并MET扩增在初治患者中的发生率为1%-5%；作为继发驱动基因，在第一、二代EGFR-TKIs耐药后的发生率为4%-22%，在第三代EGFR-TKIs耐药后的发生率为15%-30%；作为共同驱动基因，在EGFR阳性初治患者中的发生率为2%-10%^[19]^。EGFR突变与MET基因扩增/过表达的共存降低了对EGFR-TKIs的敏感性，这可能是导致一线EGFR-TKIs单药治疗产生原发性耐药的关键。同时存在的原发性MET基因扩增和/或过表达与EGFR-TKIs单药治疗的进展时间缩短有关。临床研究^[20]^也表明，存在MET基因扩增/过表达的EGFR突变晚期NSCLC患者，在接受一线EGFR-TKIs单药治疗时临床疗效较差。临床医生可根据患者的经济条件、体力状况评分及病情进展情况选择合适的治疗模式，对于疾病进展迅速、体力状况较好的患者一线优选化疗以快速控制病情；而对于无法耐受化疗、病情发展相对缓慢或基础疾病较多的高龄患者，一线可尝试选择TKIs靶向治疗。不同研究对于定义MET扩增的阈值略有差异，有文献报道^[21]^可选择MET基因拷贝数与该基因所在的第7号染色体着丝粒（centromeric probe for chromosome 7, CEP7）的比值进行判读：MET/CEP7<1.8为阴性；1.8-2.2为低表达；2.2-5.0为中表达；≥5.0为高表达。一般而言，MET扩增程度越高，合并其他突变比例越低，驱动性越高。故对于继发MET高水平扩增的肺癌患者，可能会成为靶向联合治疗的优势人群。

在2024年9月7日至10日举办的世界肺癌大会（World Conference on Lung Cancer, WCLC）上，广东省人民医院杨衿记教授报告了CTONG 2008（FLOWERS）（摘要号：PL04.07）的主要研究结果^[22]^。这是一项探索奥希替尼联合赛沃替尼（一种高选择性MET抑制剂）作为一线治疗在EGFR突变且MET异常的晚期NSCLC患者中的疗效和安全性的研究。该研究共纳入44例患者中存在EGFR敏感突变和MET异常（包括MET扩增和/或MET过表达），其中23例接受奥希替尼单药治疗，21例接受奥希替尼联合赛沃替尼治疗。中位随访时间为8.2个月，奥希替尼单药治疗组中位PFS为9.3个月，奥希替尼联合赛沃替尼治疗组中位PFS为19.6个月（为单药组的2倍以上），为今后EGFR突变合并原发MET扩增的肺癌患者的治疗选择提供了依据。

在EGFR突变继发MET扩增研究领域，TATTON研究^[23]^也为我们带来了鼓舞人心的数据。这是一项旨在评估赛沃替尼（600 mg/300 mg口服，每日一次）联合奥希替尼（80 mg口服，每日一次）治疗既往接受过EGFR-TKIs治疗的EGFR突变阳性且MET扩增的晚期NSCLC患者的初步有效性和安全性的研究。研究结果显示耐药后患者接受赛沃替尼联合奥希替尼治疗后可再获得5-12个月的获益，特别是MET拷贝数≥10的患者获益更为显著。本例患者MET拷贝数达到11，属于MET抑制剂优势治疗人群，这可能也是患者接受赛沃替尼治疗后再次获得持续获益的原因。此外，在SAVANNAH II期研究^[24]^中，赛沃替尼联合奥希替尼治疗的初步分析结果显示免疫组化（immunohistochemistry, IHC）90+和/或荧光原位杂交（fluorescence in situ hybridization, FISH）10+患者具有更佳获益，中位PFS为7.1个月，再次证实MET-TKIs在克服EGFR-TKIs耐药中的重要地位。

综上所述，EGFR-TKIs在肺鳞癌患者中的疗效不如肺腺癌患者，往往在短时间内出现病情进展，故多数仍以含铂类药物为基础的化疗作为首选方案。不过具有某些特定因素的患者仍可从EGFR-TKIs治疗中获益，如亚裔女性、病理含腺癌成分、活检为小样本、无吸烟史等，这表明患者的临床特征对疗效存在影响，也为一部分无法耐受标准治疗方案的患者带来生存获益。EGFR-TKIs耐药后合并继发MET扩增目前指南中并无标准治疗方案，许多针对MET扩增的靶向药物如赛沃替尼、特泊替尼、谷美替尼等与第三代EGFR-TKIs联合应用的个体化治疗在临床中已取得一定疗效，且国内外正在开展多项临床试验进行疗效及安全性评价，期待研究结果能提供高级别的循证学依据，为这类肺癌患者带来新的生存希望。
